# Co-feeding Transmission and Its Contribution to the Perpetuation of the Lyme Disease Spirochete Borrelia afzelii

**DOI:** 10.3201/eid0812.010519

**Published:** 2002-12

**Authors:** Dania Richter, Rainer Allgöwer, Franz-Rainer Matuschka

**Affiliations:** * Humboldt-University of Berlin, Berlin, Germany

**Keywords:** transmission, co-feeding, Lyme disease, Borrelia afzelii, Ixodes ricinus

## Abstract

To determine whether direct passage of spirochetes between co-feeding vector ticks contributes to the likelihood that the Lyme disease spirochete Borrelia afzelii will perpetuate in nature, we compared the effects of time and space on transmission efficiency between simultaneously feeding ticks. The likelihood of co-feeding transmission increases with duration of attachment of the infecting tick. Co-feeding transmission becomes less efficient as distance from the infecting tick increases. Approximately 6 times as many ticks acquire infection when feeding on infected mice than when co-feeding with infected ticks. Both subadult stages of the wood tick Ixodes ricinus infrequently co-infest mice and voles in nature; on approximately 1 in 20 small rodents, larvae co-feed with spirochete-infected nymphs. Because only 1 in 100 larvae in nature appear to acquire spirochetal infection when co-feeding with infected nymphs, perpetuation of B. afzelii depends largely on horizontal transmission of such pathogens from previously infected mice to noninfected larvae.

Risk of Lyme disease generally is associated with the presence of ticks of the Ixodes ricinus complex and with particular rodents that support dense spirochetal infections. Lyme disease spirochetes migrate through the skin of their vertebrate hosts, where they are imbibed by feeding vector ticks; infectivity increases as the spirochetes multiply and disseminate. Rodent hosts are most infectious to ticks approximately 2 weeks after they have acquired infection; the hosts then infect virtually all ticks feeding on them (1,2). These pathogens may also pass directly from infected to noninfected ticks while the ticks are feeding simultaneously in close proximity and before the spirochetes have disseminated throughout the skin of their hosts (3–5). Thogotovirus, another tick-borne agent, can pass directly from infectious to noninfected ticks, even in a nonviremic host (6). This direct tick-to-tick mode of transmission may be crucial in perpetuating tick-borne encephalitis virus because ticks could acquire infection when feeding on immune hosts (7). Even vertebrate hosts without a systemic infection might, thereby, infect vector ticks (6).

Time and space may limit the efficiency of spirochetal transmission between co-feeding ticks. Although Lyme disease spirochetes appear to be cotransmitted efficiently when noninfected and infected ticks feed simultaneously on the same ear or other body part of a mouse, no larvae become infected when attached to the opposite ear or to the animal’s back (3–5). Only a few noninfected ticks become infected when permitted to attach at random to an animal that is serving simultaneously as host to spirochete-infected ticks, even when numerous infected ticks have been applied (4,5). A temporal effect may similarly limit the efficiency of co-feeding transmission because spirochetes are deposited in the skin only after the infecting tick has been feeding for approximately 2 days (8), and such ticks detach 1 or 2 days later. In the event of disseminated infection in the tick, transmission would occur somewhat more rapidly. The combined role of time and distance in the efficiency of co-feeding transmission, however, has not been rigorously examined.

Direct passage of spirochetes between co-feeding vector ticks may contribute to the likelihood that Lyme disease spirochetes will perpetuate in nature. To examine this hypothesis, we evaluated the effects of interfeeding distance and time interval between infected and noninfected ticks on the efficiency of co-feeding transmission. In particular, we compared the effects of time and space on transmission efficiency between simultaneously feeding ticks. In addition, we determined the frequency of infection in ticks randomly feeding on mice that were simultaneously parasitized by an infectious tick and compared that estimate with the frequency of infection for ticks feeding on mice that had previously served as host to an infectious tick. Lastly, we estimated the frequency of larvae co-feeding with infected nymphal ticks on rodents in disease-endemic foci of transmission.

## Materials and Methods

Outbred hairless mice (Mus musculus, SKH-1 strain), originally purchased from Charles River Deutschland (Sulzfeld, Germany), were bred and maintained in the laboratory. I. ricinus ticks were infected by Borrelia afzelii spirochetes. Virtually all such nymphal ticks contained spirochetes. The strain of B. afzelii originated from a naturally infected nymphal I. ricinus tick collected from vegetation in suburban Berlin (9). We have previously characterized this strain and maintained it by serial passage through jirds (Meriones unguiculatus) and ticks. Noninfected laboratory-reared larval I. ricinus were derived from adults in their third generation of continuous laboratory rearing by using noninfected hosts. To confirm that such larvae were free of spirochetes, pooled samples of each batch were routinely analyzed by polymerase chain reaction (PCR) amplification of a fragment of the 16S rRNA gene (see below).

In experiments designed to determine how readily spirochetes pass between co-feeding ticks, a single B. afzelii–infected nymph was permitted to attach between the shoulders of each mouse. Approximately 100 noninfected larvae were brushed onto each mouse to attach in close proximity to the feeding nymph and also at 1 and 2 cm ± 0.2 cm from the site of nymphal attachment. Any larvae that had attached elsewhere on the hairless mouse were promptly removed with forceps. Larval ticks were placed on hosts either at the time of nymphal attachment or at 24, 48, or 72 hours thereafter. Three mice were used for each time point. Each infested mouse was kept individually in a wire-mesh restraining tube suspended over water until the mouse was free of all ticks. Mice were fed standard laboratory chow and apple slices; the contents of the water pan were changed twice a day. When larvae were engorging rapidly and had become almost replete, generally approximately 48 hours after attachment, they were carefully removed with forceps and transferred to separate tubes according to their distance from the nymph. The tubes were half-filled with water-saturated plaster of paris. Engorged larvae were kept at 20±2°C under a light-dark regimen (16:8) until they molted. To confirm successful infection in each mouse, these hosts underwent xenodiagnosis with noninfected larval ticks at 2 weeks after the infected nymphal ticks had been permitted to feed. After engorged larvae had molted to the nymphal stage, at least 10 specimens from each group were examined for the presence of spirochetes by dark-field microscopy. If the apparent rate of infection in a group was <10%, the remaining ticks were analyzed by PCR.

For PCR, the body of a tick was opened, and the contained mass of soft tissue was dissected out in physiologic saline and transferred to a tube containing 180 μL lysis buffer (ATL Tissue Lysis Buffer, Qiagen, Hilden, Germany) and 20 μL proteinase K (600 mAU/mg). Midguts were lysed at 56°C overnight. DNA was extracted by using the QIAamp DNA Mini Kit (Qiagen), according to the manufacturer’s instructions, eluted with 50 μL elution buffer, and stored at –20°C until PCR was performed.

DNA of Lyme disease spirochetes was detected by amplifying a 650-nucleotide segment of the gene encoding the 16S rRNA. To increase sensitivity for detecting spirochetal DNA in ticks, we used nested PCR. Aliquots of DNA suspensions (2 μL) were diluted to 50 μL by using 200 μm of each deoxynucleoside triphosphate, 1.5 mM MgCl2, 0.5 U Taq polymerase (Qiagen) as well as 15 pmol of the outer primer pair and PCR buffer supplied with the Taq polymerase. We used the following primer sequences of the 16S rRNA gene (10): outer primers (5) 16S1A – CTAACGCTGGCAGTGCGTCTTAAGC and 16S1B – AGCGTCAGTCTTGACCCAGAAGTTC (positions 36–757). The mixture was placed in a thermocycler (PTC 200, MJResearch, Biozym, Germany), heated for 1 min at 94°C, and subjected to 30 cycles each, including a 20-sec denaturation at 94°C, a 20-sec annealing reaction at 63°C, and a 40-sec extension at 72°C. A final extension for 2 min at 72°C was added to the last cycle. After the first amplification with the outer set of primers, 2 μL of the amplification product was transferred to a fresh tube containing 48 μL of the reaction mixture described above, except that 2.5 mM MgCl2 and 20 pmol of the inner primer pair: (5) 16S2A–AGTCAAACGGGATGTAGCAATAC and 16S2B – GGTATTCTTTCTGATATCAACAG (positions 66–720) were used. This mixture was subjected to 35 amplification cycles by using the cycle conditions described above, except that the annealing reaction was performed at 56°C and the extension reaction lasted 30 sec. DNA was extracted, reaction vials were prepared for amplification, templates were added, and products underwent electrophoresis in separate rooms. For comparison, each PCR amplification series included DNA from a laboratory-reared nymph that had fed in its larval stage on B. burgdorferi s.s.–infected jirds. In each fifth reaction mix, water was added instead of extracted DNA to serve as a negative control. PCR products were detected by electrophoresis in a 1.5% agarose gel stained with ethidium bromide.

To determine the frequency of subadult tick stages coinfesting rodents in nature, we captured small mammals in live traps (Longworth Scientific Instruments, Abingdon, U.K.) baited with apple, grain, and cotton. Rodents were captured in two sites in southwestern Germany (Kappel and Tübingen) and one in adjacent France (Petite Camargue, Alsacienne) during April through October of 1993–1995. Each rodent was caged over water until all ticks had detached. The contents of the water pan were inspected twice daily, and ticks were promptly removed, counted, and identified. Rodents were released at the point of capture.

To determine the prevalence of Lyme disease spirochetes in questing nymphal ticks in these sites, such ticks were collected once a month during April through October of 1993–1995 by means of a flannel flag dragged through brushy vegetation. The ticks were confined in screened vials and stored at 10°C±1°C until they were identified as to stage and species and examined for spirochetes. Field-collected ticks were dissected and their midguts examined for spirochetal infection by dark-field microscopy.

## Results

First, we determined whether the duration of nymphal attachment before larval attachment affects the likelihood of spirochetal transmission between co-feeding ticks. Few larvae acquired infection unless they had attached some time after the infecting nymph had attached (Table 1). Some larvae became infected when they were placed on the host after 2 days; more larvae became infected when they began to feed on the 3rd day of nymphal attachment, when the infecting nymph was becoming replete and had begun to detach. The likelihood of co-feeding transmission therefore increases with duration of attachment of the infecting tick.

**Table 1:** Spirochetal infection in larval Ixodes ricinus ticks that fed on mice during the period of attachment of a single Borrelia afzelii–infected nymph and that fed at specified distances from the infecting nympha

Duration of nymphal attachment before larvae attached (days)	Distance between nymph and larvae (cm)	Infection in co-feeding larvae	
		No. examined	% infected
0	Nil	68	0
	1	83	0
	2	51	0
1	Nil	125	1.6
	1	74	0
	2	124	0
2	Nil	67	29.9
	1	87	5.7
	2	54	1.9
3	Nil	94	55.3
	1	82	25.6
	2	160	6.3

aEach feeding sequence was replicated three times.

We then evaluated the effect of interfeeding distance on the efficiency of co-feeding transmission. About half of the larvae became infected when they fed virtually in contact with the infecting nymph and when the nymph had become replete (Table 1). Fewer than a quarter became infected when feeding 1 cm from the infecting tick and even fewer at 2 cm distance. These findings suggest that co-feeding transmission becomes less efficient as distance from the infecting tick increases.

The efficiency of transmission between ticks feeding randomly, but simultaneously, on the same host was compared to the frequency of infection in ticks feeding on mice that had been infected 2 weeks earlier. In the simultaneously feeding ticks, cohorts of noninfected larvae were permitted to attach to mice 3 days after one spirochete-infected nymph was permitted to attach, and just before it had become replete. Many fewer simultaneously feeding ticks than sequentially feeding ticks acquired infection in this experiment (Table 2). Approximately 6 times as many ticks acquired infection in the course of feeding on infected mice than when co-feeding with infected ticks.

**Table 2:** Spirochetal infection in larval Ixodes ricinus ticks that fed randomly on bodies of mice beginning at 3 days and 14 days after a single Borrelia afzelii–infected nymph had begun to feed

Duration of nymphal attachment before larvae attached (days)	Infection in larvae	
	No. examined	% infected
3	88	13.6
14	82	85.4

The likelihood that larval and nymphal ticks might acquire spirochetal infection by co-feeding in nature was established by analyzing the distribution of subadult ticks on rodents captured in endemic foci of transmission and determining the prevalence of Lyme disease spirochetes in questing nymphs. Larvae, but no nymphs, were found on approximately two thirds of yellow-necked mice (Apodemus flavicollis), wood mice (A. sylvaticus), and bank voles (Clethrionomys glareolus) (Table 3). Nymphs and larvae coinfested about a fifth of mice and even fewer voles. Two thirds of garden dormice (Eliomys quercinus), however, were coinfested by larvae and by nymphs. Of the nymphal ticks questing in these sites, approximately one quarter (26.4%) were infected by Lyme disease spirochetes. In contrast to the relationship in garden dormice, both subadult stages of the wood tick infrequently coinfested mice and voles; in nature, larvae co-feed with spirochete-infected nymphs only on approximately 1 in 20 small rodents.

**Table 3:** Proportion of captured rodent hosts infested by larval and nymphal Ixodes ricinus ticks, southwestern Germany and Alsace

Hosts		% hosts infested by			
Kinda	No.	None	Larvae alone	Nymphs alone	Larvae and nymphs
Af	215	12.1	65.1	2.3	20.5
As	128	14.1	62.5	0.8	22.7
Cg	183	25.1	60.1	0.5	14.2
Eq	66	6.1	27.3	1.5	65.2

aYellow-necked mice (Apodemus flavicollis [Af]), wood mice (A. sylvaticus [As]), bank voles (Clethrionomys glareolus [Cg]), and garden dormice (Eliomys quercinus [Eq]).

## Discussion

The experimental demonstration that tick-borne pathogens could perpetuate in nature in the absence of reservoir hosts that develop systemic infections (6) transformed a previously central epidemiologic concept. Thereby, a reservoir host, used in the sense of the alternative to the vector host, need not support the dissemination of the pathogen. Even a virus-immune vertebrate host permits passage of that virus between simultaneously feeding vector ticks (7). The concept applies similarly to Lyme disease spirochetes transmitted by ticks feeding in close proximity to each other on competent rodent hosts (3–5) and on spirochete-incompetent sheep (11). At least in the laboratory, diverse pathogens can pass directly between vector ticks.

The efficiency of co-feeding transmission may depend on interfeeding distance. In the case of Thogotovirus, distance appears to make little difference. The virus readily passes between ticks that are feeding on guinea pigs as far as 160 mm apart, and these hosts remain nonviremic (12). Co-feeding transmission of tick-borne encephalitis virus, in contrast, is more efficient if ticks feed in close proximity than if separated by a distance of 1 cm. Langerhans cells appear to aid transmission (13). The ability of Lyme disease spirochetes to pass between co-feeding vector ticks is less pronounced. Although spirochetes readily pass between ticks confined in the same feeding chamber, none do so when the infected ticks feed on the back of a mouse while noninfected ticks are feeding on the mouse’s head (3), when attached to different ears of a jird (5), or when the distance separating the infected from the noninfected ticks is 3 cm (4). We found that the critical distance between the co-feeding pair was approximately 1 cm. Although an occasional tick might become infected by B. afzelii over a distance of 2 cm, transmission efficiency falls precipitously as the distance between co-feeding ticks approaches 1 cm.

Transmission efficiency also has a temporal component. In contrast to virus particles, which are present in the salivary glands at the time of attachment (14), North American Lyme disease spirochetes as well as B. afzelii are injected into the skin of a vertebrate host only after the infecting tick has been attached for more than a day (8, unpub. data). Because nymphal I. ricinus remain attached for approximately 3 days and larvae 1 day less, the co-feeding window remains open only briefly. A larva could not ingest spirochetes if it attached at the same time as did the infected co-feeding nymph. This scenario conforms well to our observations. Other Ixodes vector ticks feed approximately 1 day longer than do subadult I. ricinus, a pattern that explains why some larvae, described in other studies, became infected when permitted to attach at the same time as the infecting nymph (4,5). The relationship between time and distance is particularly complex because of the ability of these spirochetes to move rapidly through skin (15). They disseminate through this matrix only after the infecting tick has become replete and has detached (1). Indeed, we found that the efficiency of co-feeding transmission correlates inversely with distance between the feeding pair. In general, less than 14% of randomly attached ticks acquire infection by co-feeding transmission.

The number of co-feeding larval and nymphal ticks appears to affect the efficiency of transmission of Lyme disease spirochetes. No spirochetes are transmitted between co-feeding ticks when natural densities of subadult I. scapularis ticks infest the white-footed mouse (Peromyscus leucopus) (16). In contrast, up to 5% of 200 larvae acquire B. burgdorferi s.s., when co-feeding with as many as 40 infected nymphal I. scapularis ticks on the North American reservoir rodent (16). In nature, however, this density of subadult ticks on murine hosts is unlikely (16). Even fewer infected nymphal ticks generally feed on the European reservoir rodents (17). Although efficiency of transmission of Lyme disease spirochetes increases with density of co-feeding ticks, such tick densities are extremely rare in nature.

A synthetic model has recently been employed to estimate the overall contribution of co-feeding transmission to the intrinsic rate of natural increase (R0) of populations of Lyme disease spirochetes (18). This model is based on major assumed parameters that include 1) competence of the vector and reservoir hosts, combined with duration of infectivity, and 2) proportion of feeding ticks that acquire infection, combined with the effect of distance between co-feeding ticks. Although conventional “systemic” transmission would be far more important than nonsystemic tick-to-tick transmission in the case of Lyme disease spirochetes, these considerations suggest that “any host that feeds large numbers of ticks should now be considered a candidate as an amplifying host”(18). Reservoir-incompetent vertebrate hosts appear to contribute an important component to the force of transmission.

We found that 1 cm appears to be the critical distance separating infectious from susceptible ticks that inhibits transmission of B. afzelii between simultaneously feeding ticks. If one assumes that infected reservoir rodents remain infectious throughout their lives, some 85% of larval Ixodes ticks acquire infection from rodents when feeding “sequentially,” i.e., on hosts that had previously been infected by nymphal ticks. In contrast, less than 14% of larvae do so when they feed “simultaneously” with infected nymphs. In nature, about 20% of small rodents carry both subadult stages (19 and current study), and spirochetes infect approximately 26% of these nymphs. By simple multiplication, then, less than 1% of vector ticks (14 x 20 x 26 = 0.73%) would acquire spirochetal infection during co-feeding, and even fewer would become infected by B. afzelii. This calculation corresponds closely to our observation of spirochetal infection in larval ticks that had attached, in nature, to hosts that do not support spirochetal infection (20). The previous theoretical estimate suggests that six times as many infected vector ticks derive from larvae that fed on spirochete-infected hosts than would result if they co-fed with infected ticks on noninfected hosts (18). Our combined experimental and field-derived evidence, however, indicates that the transmission efficiency between sequentially feeding ticks exceeds that between co-feeding ticks by a ratio of at least 100:1 (85/0.73=116). Perpetuation of the Lyme disease spirochete B. afzelii, therefore, would depend largely on persistent dissemination of these pathogens throughout the skin of the competent vertebrate hosts on which the vector ticks mainly feed.

## References

[1] Shih C-M, Pollack RJ, Telford SR, Spielman A. Delayed dissemination of Lyme disease spirochetes from the site of deposition in the skin of mice. J Infect Dis. 1992;166:827–31. 1527418 10.1093/infdis/166.4.827

[2] Donahue JG, Piesman J, Spielman A. Reservoir competence of white-footed mice for Lyme disease spirochetes. Am J Trop Med Hyg. 1987;36:92–6. 3812887 10.4269/ajtmh.1987.36.92

[3] Gern L, Rais O. Efficient transmission of Borrelia burgdorferi between cofeeding Ixodes ricinus ticks (Acari: Ixodidae). J Med Entomol. 1996;33:189–92. 8906929 10.1093/jmedent/33.1.189

[4] Sato Y, Nakao M. Transmission of the Lyme disease spirochete, Borrelia garinii, between infected and uninfected Ixodes persulcatus during cofeeding on mice. J Parasitol. 1997;83:547–50. 10.2307/3284432 9194849

[5] Patrican LA. Acquisition of Lyme disease spirochetes by cofeeding Ixodes scapularis ticks. Am J Trop Med Hyg. 1997;57:589–93. 9392600 10.4269/ajtmh.1997.57.589

[6] Jones LD, Davies CR, Steele GM, Nuttall PA. A novel mode of arbovirus transmission involving a nonviremic host. Science. 1987;237:775–7. 10.1126/science.3616608 3616608

[7] Labuda M, Kozuch O, Zuffová E, Elecková E, Hails RS, Nuttall PA. Tick-borne encephalitis virus transmission between ticks cofeeding on specific immune natural rodent hosts. Virology. 1997;235:138–43. 10.1006/viro.1997.8622 9300045

[8] Piesman J, Mather TN, Sinsky RJ, Spielman A. Duration of tick attachment and Borrelia burgdorferi transmission. J Clin Microbiol. 1987;25:557–8. 3571459 10.1128/jcm.25.3.557-558.1987 PMC265989

[9] Matuschka F-R, Schinkel TW, Spielman A, Richter D. Failure of Ixodes ticks to inherit Borrelia afzelii infection. Appl Environ Microbiol. 1998;64:3089–91. 9687480 10.1128/aem.64.8.3089-3091.1998 PMC106822

[10] Ohlenbusch A. Beiträge zur Diagnostik und Pathogenese der Lyme-Borreliose und zur Transmission des Erregers Borrelia burgdorferi. Göttingen, Germany: Cuvillier Verlag; 1996.

[11] Ogden NH, Nuttall PA, Randolph SE. Natural Lyme disease cycles maintained via sheep by co-feeding ticks. Parasitology. 1997;115:591–9. 10.1017/S0031182097001868 9488870

[12] Jones LD, Nuttall PA. Non-viraemic transmission of Thogoto virus: influence of time and distance. Trans R Soc Trop Med Hyg. 1989;83:712–4. 10.1016/0035-9203(89)90405-7 2617637

[13] Labuda M, Austyn JM, Zuffova E, Kozuch O, Fuchsberger N, Lysy J, Importance of localized skin infection in tick-borne encephalitis virus transmission. Virology. 1996;219:357–66. 10.1006/viro.1996.0261 8638401

[14] Nuttall PA, Jones LD, Labuda M, Kaufman WR. Adaptations of arboviruses to ticks. J Med Entomol. 1994;31:1–9. 8158611 10.1093/jmedent/31.1.1

[15] Kimsey RB, Spielman A. Motility of Lyme disease spirochetes in fluids as viscous as the extracellular matrix. J Infect Dis. 1990;162:1205–8. 2230247 10.1093/infdis/162.5.1205

[16] Piesman J, Happ CM. The efficacy of cofeeding as a means of maintaining Borrelia burgdorferi: a North American model system. J Vector Ecol. 2001;26:216–20. 11813659

[17] Matuschka F-R, Fischer P, Musgrave K, Richter D, Spielman A. Hosts on which nymphal Ixodes ricinus most abundantly feed. Am J Trop Med Hyg. 1991;44:100–7. 1996733 10.4269/ajtmh.1991.44.100

[18] Randolph SE, Gern L, Nuttall PA. Co-feeding ticks: epidemiological significance for tick-borne pathogen transmission. Parasitol Today. 1996;12:472–9. 10.1016/S0169-4758(96)10072-7 15275266

[19] Randolph SE, Miklisova D, Lysy J, Rogers DJ, Labuda M. Incidence from coincidence: patterns of tick infestations on rodents facilitate transmission of tick-borne encephalitis virus. Parasitology. 1999;118:177–86. 10.1017/S0031182098003643 10028532

[20] Matuschka F-R, Heiler M, Eiffert H, Fischer P, Lotter H, Spielman A. Diversionary role of hoofed game in the transmission of Lyme disease spirochetes. Am J Trop Med Hyg. 1993;48:693–9. 8517488 10.4269/ajtmh.1993.48.693

